# Comparative Genome Analysis of Uropathogenic *Morganella morganii* Strains

**DOI:** 10.3389/fcimb.2019.00167

**Published:** 2019-05-22

**Authors:** Leyla Minnullina, Daria Pudova, Elena Shagimardanova, Leyla Shigapova, Margarita Sharipova, Ayslu Mardanova

**Affiliations:** ^1^Laboratory of Microbial Biotechnology, Institute of Fundamental Medicine and Biology, Kazan (Volga region) Federal University, Kazan, Russia; ^2^Laboratory of Extreme Biology, Institute of Fundamental Medicine and Biology, Kazan (Volga region) Federal University, Kazan, Russia

**Keywords:** *Morganella morganii*, uropathogens, genome, virulence-related genes, prophages, hemolysins

## Abstract

*Morganella morganii* is an opportunistic bacterial pathogen shown to cause a wide range of clinical and community-acquired infections. This study was aimed at sequencing and comparing the genomes of three *M. morganii* strains isolated from the urine samples of patients with community-acquired urinary tract infections. Draft genome sequencing was conducted using the Illumina HiSeq platform. The genomes of MM 1, MM 4, and MM 190 strains have a size of 3.82–3.97 Mb and a GC content of 50.9–51%. Protein-coding sequences (CDS) represent 96.1% of the genomes, RNAs are encoded by 2.7% of genes and pseudogenes account for 1.2% of the genomes. The pan-genome containes 4,038 CDS, of which 3,279 represent core genes. Six to ten prophages and 21–33 genomic islands were identified in the genomes of MM 1, MM 4, and MM 190. More than 30 genes encode capsular biosynthesis proteins, an average of 60 genes encode motility and chemotaxis proteins, and about 70 genes are associated with fimbrial biogenesis and adhesion. We determined that all strains contained urease gene cluster *ureABCEFGD* and had a urease activity. Both MM 4 and MM 190 strains are capable of hemolysis and their activity correlates well with a cytotoxicity level on T-24 bladder carcinoma cells. These activities were associated with expression of RTX toxin gene *hlyA*, which was introduced into the genomes by a phage similar to *Salmonella* phage 118970_sal4.

## Introduction

*Morganella morganii* is a gram-negative bacterium, which is a common inhabitant of the environment and intestinal tracts of humans, mammals, and reptiles (Nakao et al., 2013; Lin et al., 2015). It is also an important opportunistic pathogen, which causes a wide range of clinical and community-acquired infections (Liu et al., 2016).

Genus *Morganella*, similar to genera *Proteus* and *Providencia*, belongs to the *Proteeae* tribe (O'Hara et al., 2000), and includes species such as *M. morganii* and *M. psychrotolerans* (Emborg et al., 2006). According to the modern classification, *Morganella* is a type genus of a novel *Morganellaceae* family. This family consists of the following 8 genera: *Arsenophonus, Cosenzaea, Moellerella, Morganella, Photorhabdus, Proteus, Providencia*, and *Xenorhabdus* (Adeolu et al., 2016).

In spite of its relatively rare occurrence in clinical isolates, *M. morganii* is a frequent cause of urinary tract infections (UTIs), septicemia, and wound infections (Chen et al., 2012). *M. morganii* has occasionally been associated with pathologies of diverse localizations such as a brain abscess (Abdalla et al., 2006; Patil et al., 2012), liver abscess (Hakyemez et al., 2012; Ponte and Costa, 2015), chorioamnionitis (Liu et al., 2016), peritonitis (Atalay et al., 2010; Tsai et al., 2013), pericarditis (Nakao et al., 2013), septic arthritis (Schonwetter and Orson, 1988), rhabdomyolysis (Imataki and Uemura, 2017), necrotizing fasciitis following snakebites (Mao et al., 2016; Tsai et al., 2017), bilateral keratitis (Zhang et al., 2017), neonatal sulfhemoglobinemia (Murphy et al., 2015), and even non-clostridial gas gangrene (Ghosh et al., 2009). Erlanger et al. (2017) reported that *M. morganii* bacteremia cases are more commonly accompanied by complications or by fatal consequences in comparison to *Escherichia coli*.

UTIs are one of the most socially significant diseases. About 60% of women are estimated to acquire UTI at least once in their lifetime (Hilbert et al., 2012; Lüthje and Brauner, 2014). Although the main causative agents of UTIs are *E. coli, Klebsiella pneumoniae, Staphylococcus saprophyticus*, and *Enterococcus* spp. (Foxman, 2010; Flores-Mireles et al., 2015; Waller et al., 2018), *M. morganii* is often isolated from patients with long-term urinary catheters (Stickler, 2008, 2014). It is known that uropathogens cause infections by synthesizing a complex of virulence factors: different toxins and proteases (Hilbert et al., 2012; Welch, 2016), type I fimbriae and P-fimbriae (Kalita et al., 2014), flagella (Wiles et al., 2008), siderophores (Lüthje and Brauner, 2014), ureases (Mobley and Warren, 1987) etc. The majority of virulence factors from uropathogenic *E. coli* (UPEC) are encoded by different pathogenicity islands (PAI) (Hannan et al., 2008). Among them is a pore-forming toxin α-hemolysin (HlyA), which is expressed by more than 50% of UPEC strains and plays an important role in pathogenesis of cystitis and pyelonephritis (Ristow and Welch, 2016). HlyA is encoded by *hlyCABD* operon, which can be located on the chromosome or plasmids (Oloomi and Bouzari, 2008). α-hemolysin activity leads to ATP release and lysis of erythrocytes, lymphocytes, leukocytes, epithelial and endothelial cells of human, and other mammals (Skals et al., 2014; Chenal et al., 2015). For example, this toxin promotes NLRP3 inflammasome-mediated IL-1β release and death of human macrophages (MV Murthy et al., 2018). Although HlyA has been well-studied, some aspects of its mechanism of action and function *in vivo* presently remain unclear (Ristow and Welch, 2016). In addition, it was recently reported that about 60% of secreted HlyA can be associated to outer membrane vesicles, which was 10 fold more active than purified free toxin (Herlax et al., 2010; Thomas and Wigneshweraraj, 2014). It is known that more than 50% of clinical isolates of *M. morganii* have a hemolytic activity (Gołuszko et al., 1988; Kim et al., 2007). In 1987, Koronakis et al. (1987) reported that the genetic determinant encoding *M. morganii* secreted hemolysin was related to *hlyA* of *E. coli*. *M. morganii* hemolysin possesses similar properties to α-hemolysin, being calcium-dependent, having similar structure and function, and causing death of erythrocytes and polymorphonuclear leucocytes (Senior and Hughes, 1988; Eberspächer et al., 1990). Moreover, infection by hemolytic *M. morganii* strains has been proven to be lethal for mice (Emödy et al., 1982) and immunosuppressed humans (Kim et al., 2007). Consequently, secreted hemolysin remains a major virulence factor of *M. morganii*.

Numerous virulence-related genes have been identified in the annotated genomes of different *M. morganii* strains (Chen et al., 2012; Liu et al., 2016). As of January 2019, 55 sequenced genomes of *M. morganii* have been deposited in the Genome database, 11 of which were fully sequenced. The size of the sequenced *M. morganii* genomes averages at 3.99 Mb, with a GC content varying from 50.1 to 51.4%. However, to date, a detailed genome analysis has been carried out for only a few strains of *M. morganii* (KT, F675, and INSRALV892a) (Chen et al., 2012; Olaitan et al., 2014; Jones-Dias et al., 2016). Notwithstanding, none of them were isolated from urine.

Understanding the molecular characteristics of *M. morganii* will facilitate identification of subtle differences in the genome and pathogenicity characteristics. Here we report the genome sequences and comparative genome analysis of three *M. morganii* strains isolated from urine in UTI cases, exhibiting variances in hemolytic properties. Comparative genomic analyses which included major genome features, genomic similarities and differences among isolates, determination of the pan- and core genomes, characterization of functional gene categories using the cluster of orthologous genes (COGs) classification, and prediction of genomic islands, virulence factors, and prophages were also performed.

## Materials and Methods

### Isolation and Cultivation of Bacterial Strains

The *M. morganii* strains (MM 1, MM 4, MM 190) were isolated from the urine of a 59-year-old man, 35-year-old woman, and a 3-year-old boy, respectively, who had community-acquired urinary tract infections. Samples for MM 1 and MM 4 were collected in October 2014, whereas that of MM 190 was taken in June 2015 by the Clinical Diagnostic Laboratory Biomed (Kazan, Republic of Tatarstan, Russia). Identification of the strains was performed based on microbiological tests and mass spectrometry of protein profiles on MALDI Bio Typer (Bruker Daltonik, Germany). The Score Value >2.5 was observed in all cases, indicating a highly probable species identification. Ethical approval was not required as clinical isolates were collected and stored as part of routine clinical care.

For bacterial cultivation, LB (Lysogeny broth and Lysogeny agar) medium (1% tryptone, 0.5% yeast extract, 0.5% NaCl, pH 8.5) was used. Bacteria were grown at 37°C with aeration (Shaker Braun, Germany). The absorbance of bacterial cultures was measured at 590 nm using XMark Microplate Spectrophotometer (BioRad, Singapore).

### Hemolysis Assay

The hemolytic activity was investigated on blood agar containing 5% human erythrocytes. Plates were incubated for 24 h at 37°C. The hemolytic activity was evaluated by measuring the diameter of clear zones around the colonies, and the specific type of hemolysis (α, β, or γ) was determined by the morphology of the hemolysis zone.

To assess the accumulation of hemolysins in the medium, a 2% suspension of human erythrocytes in 0.85% NaCl was used (Senior and Hughes, 1988). LB medium was inoculated by an overnight culture to attain a 1% bacterial suspension, and incubated with aeration at 37°C. Every hour, 500 μl of culture were pelleted by centrifugation (5 min, 8,000 rpm) and the supernatant was collected in a clean tube. Next, the bacterial culture fluid was mixed with a 2% suspension of washed human erythrocytes (with 20 mM CaCl_2_) in a ratio of 9:1. Following a 30 min incubation at 37°C, samples were centrifuged at 11,600 rpm for 1 min to pellet the cells. Release of hemoglobin was determined by measuring absorbance of supernatant at 540 nm. Hemolysis level was calculated by the formula below as previously described (Rattanama et al., 2012):

(1) $$\%\text{ of hemolysis }=\;\frac{\left(OD_{540}\text{ of sample}-\;OD_{540\;}\text{of negative control}\right)}{\left(OD_{540}\text{ of positive control}-\;OD_{540}\text{ of negative control}\right)}\;\times 100$$

As negative and positive controls, a fresh culture medium without bacteria and distilled water were used, respectively.

### Cytotoxicity Assay

Human bladder carcinoma T-24 cells obtained from the Russian Cell Culture Collection (Institute of Cytology, St. Petersburg, Russia) were used as target cells for cytotoxicity assay. T-24 cells were seeded into 12-well tissue culture plates at 2 × 10^5^ cells per well in α-MEM medium with L-glutamine and without antibiotics (Biolot, Russia) containing 10% fetal bovine serum (Hyclone, USA). Cells were grown for 24 h at 37°C in 5% CO_2_. Overnight bacterial culture was pelleted, resuspended in a fresh α-MEM and added to T-24 cells to a multiplicity of infection (MOI) of about 50 bacteria per cell. Plates were incubated for 2 h at 37°C in 5% CO_2_, then α-MEM with bacteria were removed, wells were washed twice with PBS (Sigma-Aldrich, USA) and stained by 0.2% trypan blue for 5 min. Cytotoxicity of *M. morganii* strains was estimated by the number of the dead cells on an inverted microscope Axio Vert A1 (Carl Zeiss, Germany).

### Genome Sequencing

Genomic DNA of *M. morganii* strains MM 1, MM 4, and MM 190 was extracted by phenol chloroform method (Wright et al., 2017). The quantity and quality control of extracted DNA was evaluated using a NanoPhotometer P 300 (Implen, Germany) and by electrophoresis on a 1% agarose gel, respectively. DNA fragmentation was carried out using Covaris S220 (duty factor-5%, cycles per burst-200, times-105 s). Library construction was performed with a NEBNext Ultra II DNA Library Prep Kit (New England Biolabs). To check the quality of DNA fragmentation and DNA library preparation, a 2100 Bioanalyzer (Agilent Technologies, USA) and a High Sensitivity DNA kit (Agilent Technologies, USA) were used. Genome sequencing was carried out at KFU-RIKEN Laboratory (Kazan Federal University, Kazan, Republic of Tatarstan, Russia) on Illumina HiSeq2500 platform (Illumina, USA) with a 251-bp paired-end library.

### Genome Assembly, Annotation and Comparative Analysis

The raw reads of the genomes were analyzed by FastQC. To remove low-quality reads, Trimmomatic (v. 0.36) was used (Bolger et al., 2014). Assembly of processed reads was performed by SPAdes (v. 3.10.0.) (Bankevich et al., 2012), and the quality of the assembly was evaluated by Quast (Gurevich et al., 2013). To improve the quality of assembly, all reads <500 bp long and <2.0 coverage were eliminated. Codes used to pre-process and assemble the genomes can be found in Supplementary Material.

The genomes were annotated using the NCBI Prokaryotic Genomes Annotation Pipeline (Angiuoli et al., 2008). For functional annotation RAST (annotation scheme: ClassicRAST) was used (Aziz et al., 2008). Search for closely related strains was carried out using JSpeciesWS (Richter et al., 2016). To align the draft genomes relative to the reference, MeDuSa scaffolder was used (Bosi et al., 2015). Genome sequences circular comparison was performed by BRIG (Alikhan et al., 2011), BLAST tools were used for sequence alignment. To calculate the pan-genome and core-genome, and Venn diagram construction, EDGAR 2.0 was used (Blom et al., 2016). Genome loci were analyzed using ASAP (Glasner et al., 2003), Easyfig (Sullivan et al., 2011), and MAUVE (Darling et al., 2004). Phage regions were identified by PHASTER (Arndt et al., 2016) and analyzed using Virus-Host DB (Mihara et al., 2016). Pathogenicity islands were found by IslandViewer4 (Bertelli et al., 2017). GC content of distinct sequences was calculated using GC Content Calculator (https://jamiemcg.github.io/bioinf/gc.html).

### Nucleotide Sequence Accession Numbers

The draft genome sequences of *M. morganii* strains were deposited in the DDBJ/EMBL/GenBank under the accession numbers QUOO00000000, QPLM00000000, and QMKL00000000 for MM 1, MM 4, and MM 190, respectively. The versions described in this paper are versions QUOO01000000, QPLM01000000, and QMKL01000000. Additionally, the raw reads were deposited in the SRA database under the BioProject accession numbers PRJNA484881, PRJNA482068, and PRJNA478302 for MM 1, MM 4, and MM 190, respectively.

## Results and Discussion

### General Features of *M. morganii* Strains Genomes

As a result of genome sequencing, a total of 2,765,361, 2,968,804, and 2,933,802 reads for *M. morganii* MM 1, MM 4, and MM 190 were generated. These reads have been cleaned and assembled into 21, 36, and 63 scaffolds for MM 1, MM 4, and MM 190, respectively (Table 1).

**Table 1:** Genome assembly results for *M. morganii* strains.

**Arguments**	***M. morganii*** **genomes**		
	**MM 1**	**MM 4**	**MM 190**
No. of scaffolds	21	36	63
Largest contig (bp)	1132090	990763	1435078
Genome coverage	169.4x	181.8x	179.0x
N50	888743	403356	723928

The genomes were annotated using the NCBI Prokaryotic Genomes Annotation Pipeline (Table 2). The three *M. morganii* strains have a genome size of 3.82–3.97 Mb with an average GC content of 51%, which correlates well with properties of other annotated *M. morganii* genomes (https://www.ncbi.nlm.nih.gov/genome/genomes/10874?). About 4,000 genes were identified in the genomes, 96.1% of them were the protein-coding sequences (CDSs), 2.7% encoded RNAs, and 1.2% represented pseudogenes. We have determined that the genome size of *M. morganii* MM 190 was the largest among the three strains. In MM 190, we documented 3,968,545 bp-long genome with a GC content of 50.9%, containing 3,907 predicted genes. Of them, 3,745 were identified as protein-coding sequences, while 33, 75, and 4 genes encoded rRNAs, tRNAs, and noncoding RNAs, respectively. In addition, 50 genes were found to be pseudogenes. In contrast, MM 1 and MM 4 genomes consisted of 3,882,881 bp and 3,815,979 bp, respectively, with a GC content of 51.0% for both. 3,726 and 3,685 genes were annotated for MM 1 and MM 4, of which 3,587 and 3,548 were identified as CDSs, 74 and 73 as tRNA genes, 40 and 45 as pseudogenes, whereas 21 and 15 coded for rRNAs.

**Table 2:** Comparative genome features of *M. morganii* MM 1, MM 4, and MM 190.

**Features**	***M. morganii*** **genomes**		
	**MM 1**	**MM 4**	**MM 190**
Genome size (bp)	3882881	3815979	3968545
GC content (%)	51.0	51.0	50.9
No. of predicted genes	3726	3685	3907
No. of protein-coding sequences	3587	3548	3745
No. of predicted rRNAs	21	15	33
No. of predicted tRNAs	74	73	75
No. of other RNAs	4	4	4
No. of pseudogenes	40	45	50

**Figure 1:**
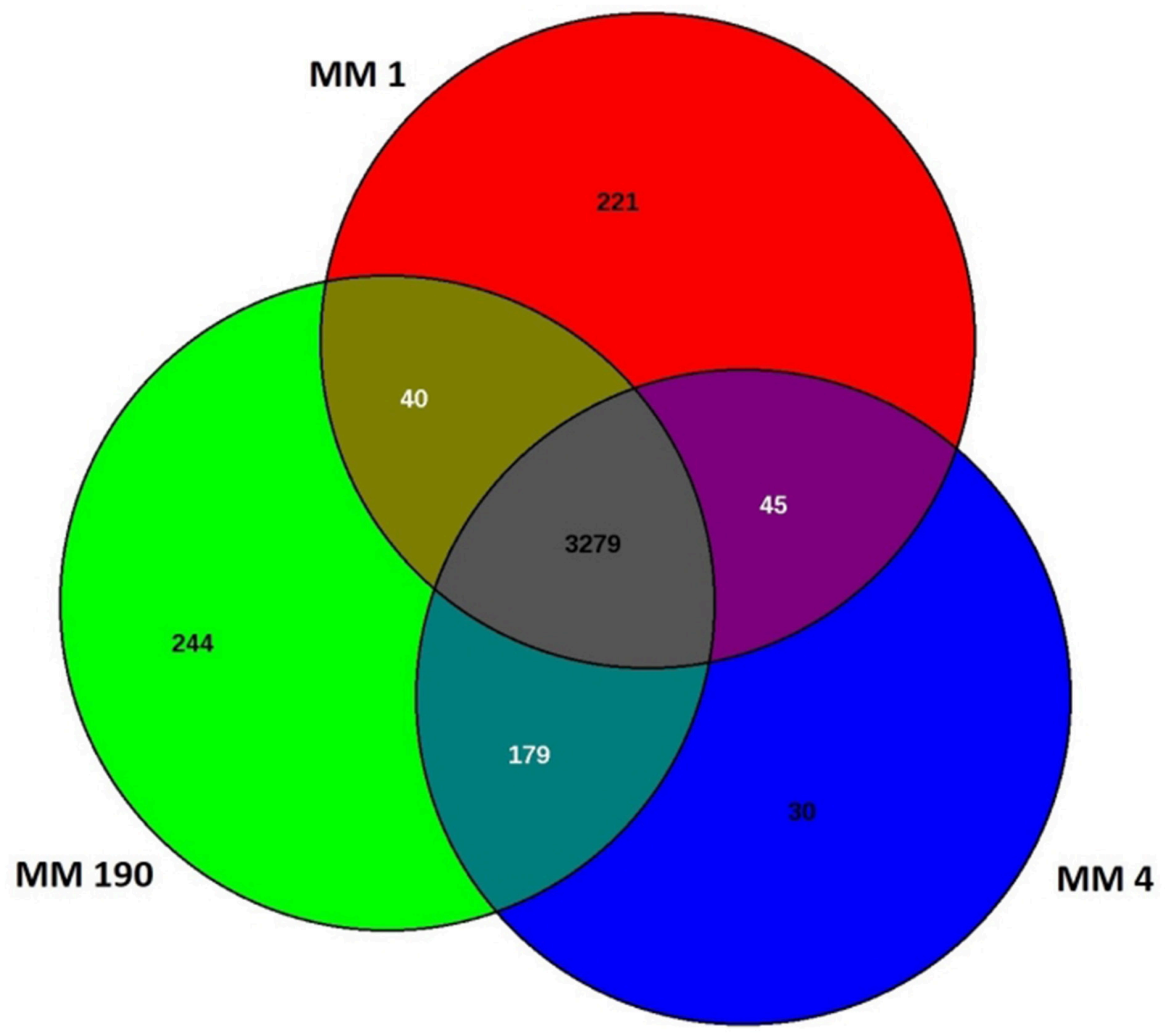
Venn diagram showing the number of shared and unique protein-coding sequences in identified *M. morganii* strains.

Comparative genome analysis of many bacterial species have demonstrated significant intra-species genome variabilities (Tettelin et al., 2008). Thus, to obtain a general assessment of the gene repertoire for studied species, it is essential to calculate the pan-genome which represents the total number of non-redundant genes, as well as core genome, which includes genes shared by all strains and encodes proteins necessary for basic biological functions. Using the EDGAR platform, we found that the pan-genome of *M. morganii* isolates comprises 4,038 genes. The core genome of MM 1, MM 4, and MM 190 strains contained a total of 3,279 protein-coding genes (Figure 1). MM 1 shared 40 and 45 genes with MM 190 and MM 4, respectively. In contrast, MM 190 and MM 4 had 179 common sequences. 221, 30, and 244 unique genes were identified in the genomes of MM 1, MM 4, and MM 190, majority of which encoded hypothetical and phage-associated proteins. We likewise calculated the pan- and core-genome of the studied strains and eleven *M. morganii* strains with whole-genome sequences, which had been deposited in the Genome database as of January 2019 (Supplementary Table 1, Supplementary Figure 1). The 14 genomes of *M. morganii* have a pan-genome consisting of 7,152 CDS and a core-genome containing 2,826 protein-coding genes.

### Genome Neighbor Identification

**Figure 2:**
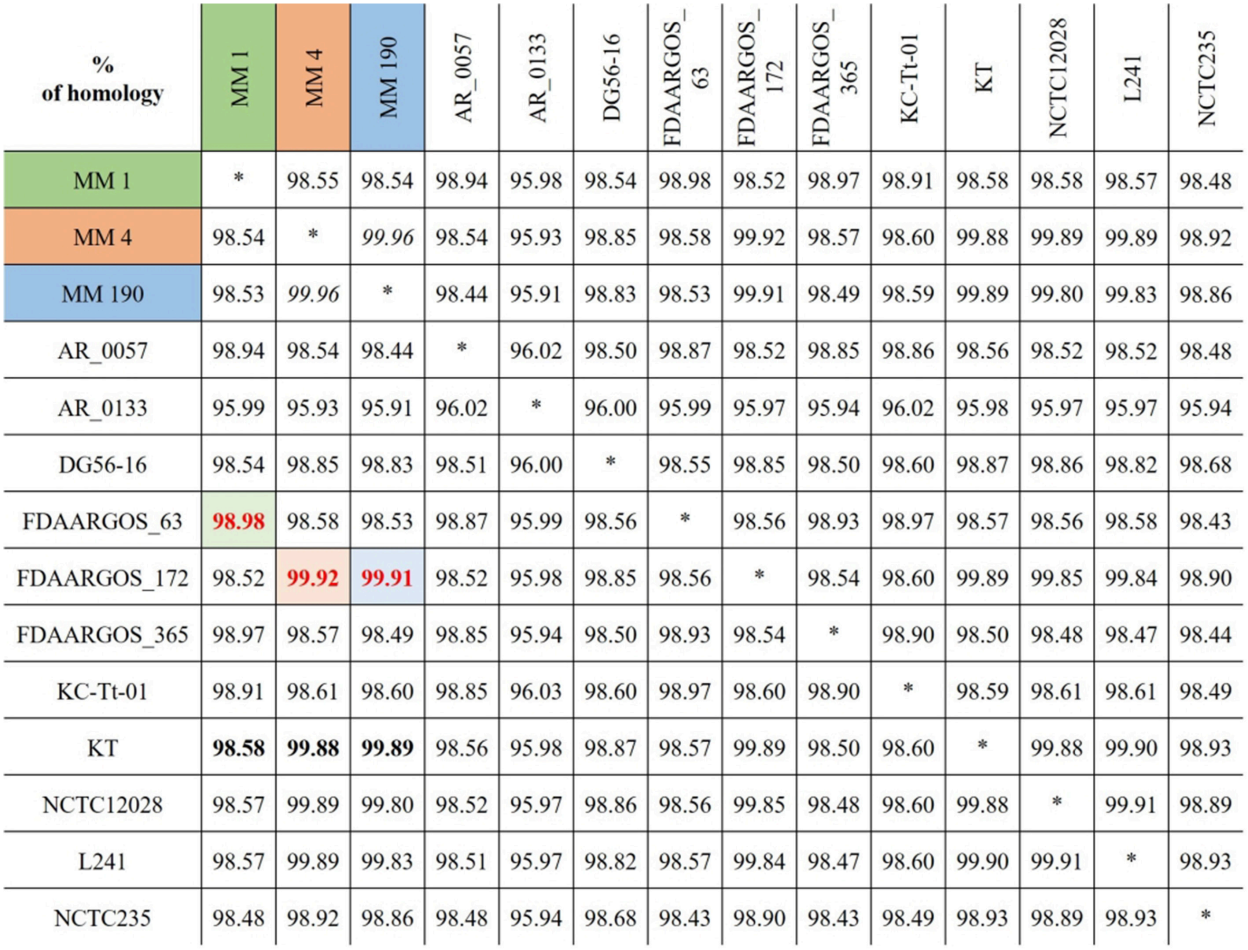
Alignment results for the genomes of identified *M. morganii* strains using JSpeciesWS (January, 2019).

To find the closest genomes of *M. morganii* MM 1, MM 4, and MM 190 strains, we considered 11 fully sequenced genomes of *M. morganii*, which were described at the previous section (Figure 2). Using JSpeciesWS, we determined that *M. morganii* FDAARGOS_63 (CP026046) was the closest neighbor of MM 1 strain, since they shared a 98.98% similarity. Conversely, MM 4 (99.92%) and MM 190 (99.91%) showed maximum homology with the *M. morganii* FDAARGOS_172 strain (CP014026). We likewise discovered that *M. morganii* KT (CP004345), which had been used as a reference strain in most previous studies, had 98.58, 99.88, and 99.89% identity with MM 1, MM 4, and MM 190, respectively. It should be noted that, while *M. morganii* FDAARGOS_172 was also obtained from urine of patient with UTI, similar to the strains we identified, the isolation source of *M. morganii* KT was human blood (Chen et al., 2012). However, *M. morganii* KT was the closest neighbor for the studied strains, when they were analyzed as a whole. Therefore, *M. morganii* KT was used as a reference in the subsequent genome analysis.

**Figure 3:**
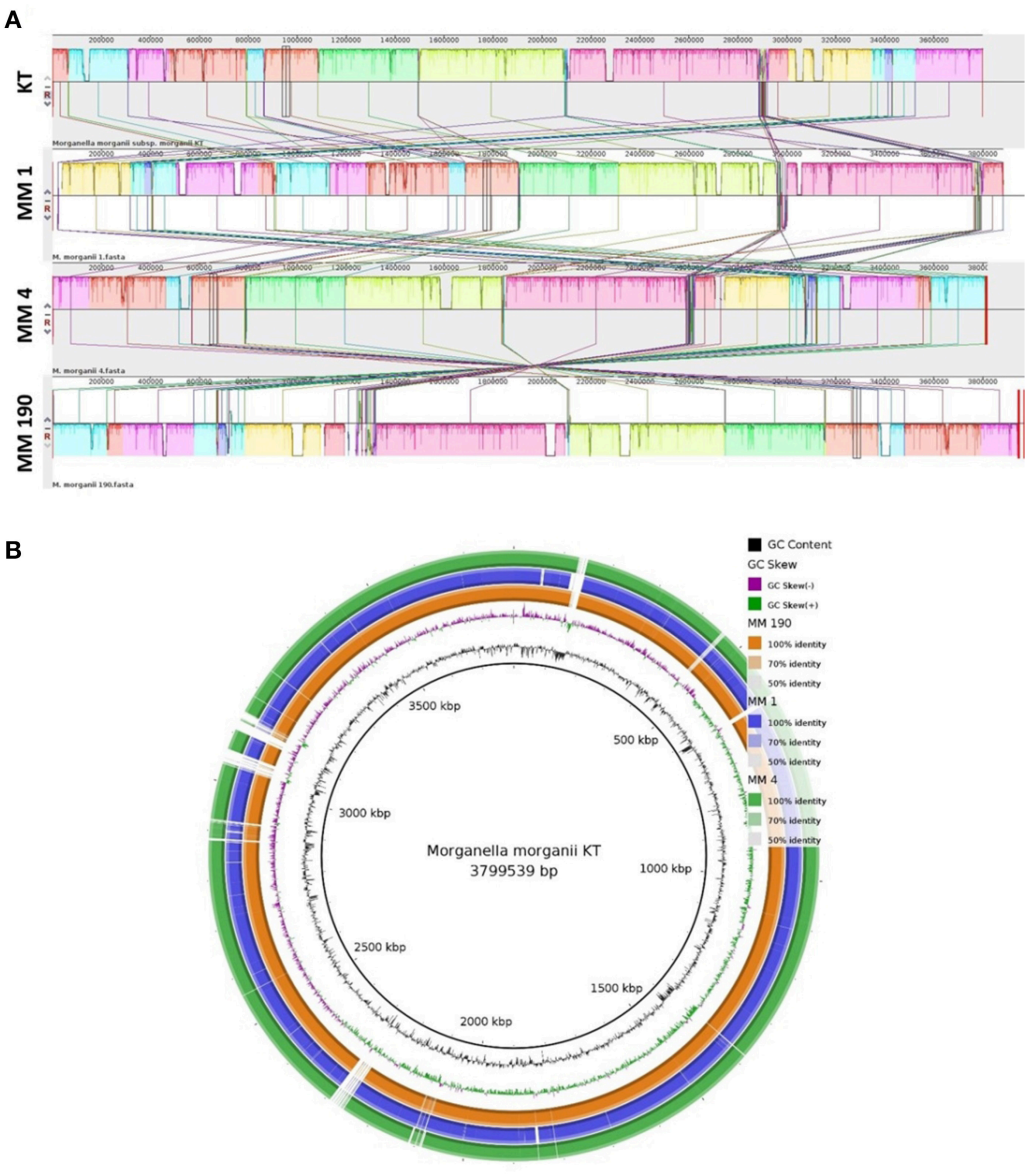
Multiple genome alignment of the genomes of *M. morganii* MM 1, MM 4, and MM 190 strains. The genome of *M. morganii* KT was used as a reference. **(A)** Genome alignment by MAUVE. The highly homologous regions are indicated by identical colors. Each block represents a similarity profile of the genome sequence. Height of profile indicates the genomic conservation level inside each block. White areas specify the unique sequences of the genome. **(B)** Circular genomic maps of *M. morganii* strains obtained using BRIG. Color saturation indicates the homology rate, blanks show the absence of similarity.

We next performed multiple genome alignment of the *M. morganii* strains in relation to *M. morganii* KT using MAUVE and BRIG (Figures 3A,B). MAUVE analysis determined that all genomes shared high content of homologous regions, however, certain loci peculiar to the *M. morganii* KT genome were also identified (Figure 3A). Circular-comparison of *M. morganii* genomes by BRIG showed that MM 1, MM 4, and MM 190 contained similar regions, which differed from the KT strain (Figure 3B). Thus, it is possible that MM 1, MM 4, and MM 190 could have a similar origin.

### Functional Annotation of Genomes

#### Coding Sequences

For detailed analysis of the genomes, the RAST annotation server was used. We found that about 40% of genes annotated by this server do not belong to any of the subsystems. In our instance, RAST found 3,580 coding sequences and 84 RNA genes in the genome of *M. morganii* MM 1, and 57% of annotated sequences belonged to subsystems (Supplementary Figure 2). We identified 3,529 CDSs and 83 RNA genes in *M. morganii* MM 4 genome and 3,711 CDSs and 84 RNA genes in *M. morganii* MM 190 genome using this server. Respectively, 57 and 55% of these genes belong to subsystem categories.

**Figure 4:**
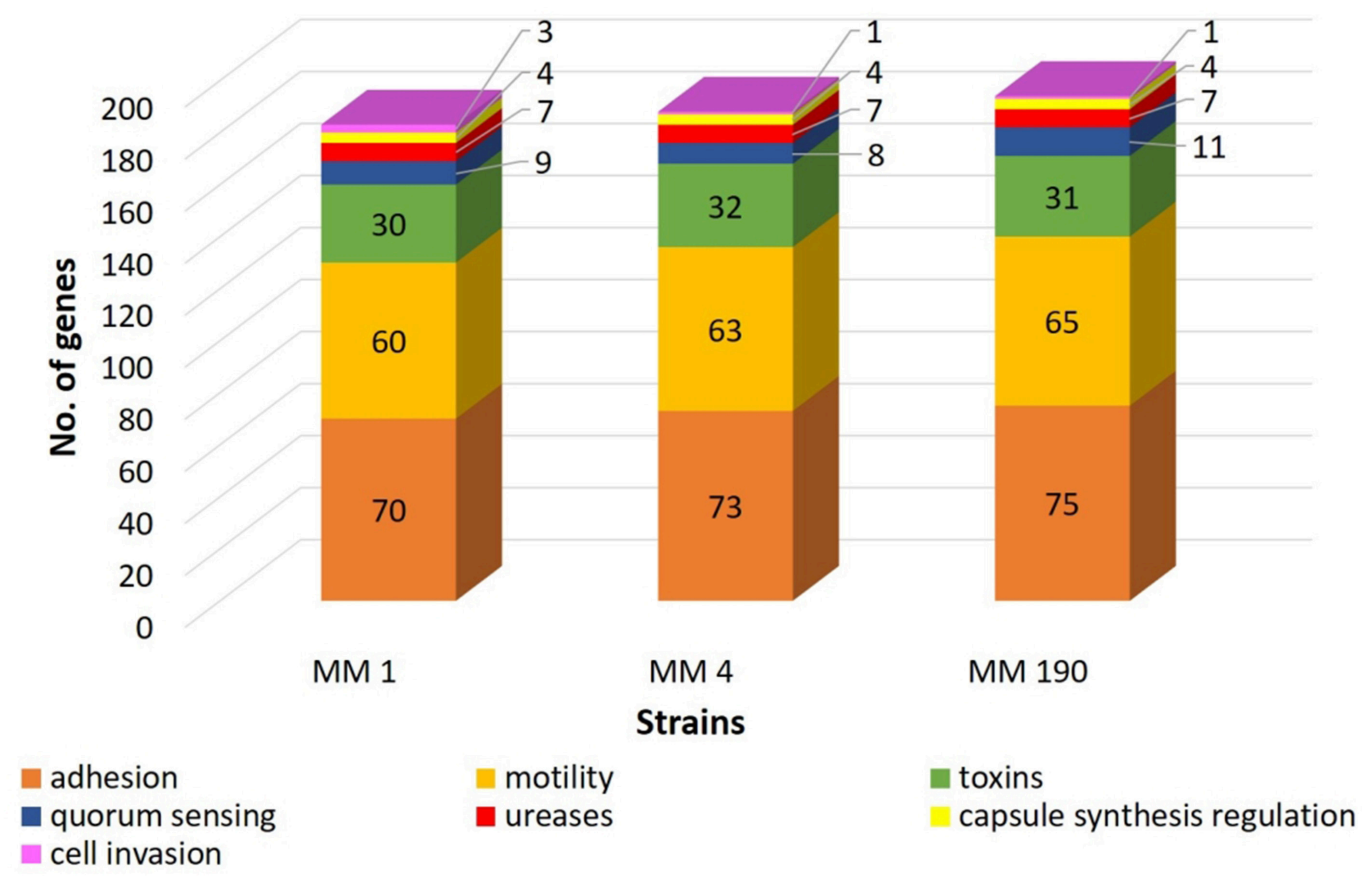
A comparison of the number of virulence-related genes in genomes of uropathogenic *M. morganii* strains obtained from annotation results of RAST and NCBI.

RAST revealed that the three annotated *M. morganii* genomes contain virulence-related genes corresponding to the following categories: “cell wall and capsule” (163–172 genes); “regulation and cell signaling” (108–110 genes), including “quorum sensing and biofilm formation” (9 genes) and “regulation of virulence” (9 genes); “motility and chemotaxis” (56 genes); “iron acquisition and metabolism” (9–19 genes); and “virulence, disease and defense” (78–79 genes). MM 4 and MM 190 strains had the same number of genes under the categories above. This correlates well with genome alignment results because they have a 99.96% of genome identity (Figure 2). We detected that RAST annotated fewer genes in comparison with NCBI tools. Hence, we used the results of both RAST and the Prokaryotic Genomes Annotation Pipeline for the identification of all virulence-related genes in the *M. morganii* genomes (Figure 4; Supplementary Table 3). Notwithstanding, there were no significant differences between the gene number of the strains.

#### Phage Regions and Pathogenicity Islands

A large quantity of pathogenic bacteria contain intact or degenerated prophage regions in their genomes (Casjens, 2003; Davies et al., 2016) majority of which code for different virulence factors (Brüssow et al., 2004). Prophage diversity is caused by high frequency of recombination with other phages, mobile elements, and genomic DNA of host bacteria. This flexibility also leads to divergence of bacterial strains within species. Thus, a large part of strain-specific DNA are associated with prophage sequences (Casjens, 2003; Zubair et al., 2015). Both intact and incomplete prophages can impart important biological properties of their host. Consequently, to fully understand bacterial genomes, the identification and analysis of their prophages are important.

Prophage sequences diversity in different *M. morganii* strains was investigated. Using PHASTER, we identified 7, 6, and 10 prophages in the genomes of MM 1, MM 4, and MM 190 strains, respectively (Supplementary Table 2). *M. morganii* MM 1 genome contains 4 intact prophages similar to mEp460 (49.7 Kb), phiO18P (34.5 Kb), HK446 (41.3 Kb), and vB_SosS_Oslo (24.5 Kb). The same number of intact prophages was determined in the genome of *M. morganii* MM 4. However, they showed resemblance with Gifsy (17.3 Kb), phiO18P (33.8 Kb), cdtl (19.2 Kb), and 118970_sal3 (12 Kb). Finally, *M. morganii* MM 190 has five intact prophages, which showed similarities with the following: Fels1 (35.3 Kb), ST64B (14.8 Kb), ENT47670 (45.2 Kb), 118970_sal3 (19 Kb), and HK446 (7.7 Kb). All genomes contain incomplete prophage homologous to *Burkholderia cenocepacia* phage BcepB1A (NC_005886, Summer et al., 2006). MM 1 and MM 4 contained intact prophage similar to *Aeromonas* virus phiO18P (NC_009542, Beilstein and Dreiseikelmann, 2008), whereas in the genomes of MM 1 and MM 190 an intact prophage possessing a 70% identity with *Enterobacteria* phage HK446 (NC_019714) was identified. Two prophage homologous to *Salmonella* phages 118970_sal4 (NC_030919) and 118970_sal3 (NC_031940, Paradiso et al., 2016) were found to be common for MM 4 and MM 190. *M. morganii* MM 190 had duplicated 118970_sal3 prophages, though one of them was incomplete. In addition, insertions of 118970_sal4 contained *hlyCABD* operon encoding an α-hemolysin homolog.

Thus, the strains contain 6-10 prophages (16 unique types of prophages for three genomes), which represent 5.4, 3.1, and 6.2% of the MM 1, MM 4, and MM 190 genomes, respectively. This is typical for enterobacteria. For instance, the *E. coli* strain K-12 contains 9 prophages which account for 3.6% of its genome (Wang et al., 2010). 5 ± 3 prophage regions have been identified in the genomes of different serovars of *Salmonella enterica* (Mottawea et al., 2018), although some of them contained up to 15 prophages per genome. This is also true for other *M. morganii* strains. For example, *M. morganii* F675 had 9 prophages (6 of them were intact), comprising 7.3% of the total genome sequence (Olaitan et al., 2014). *M. morganii* INSRALV892a contained 10 prophage regions, of which four complete prophages showed the high similarity with *Enterobacteria* phages SfV and mEp235 (Jones-Dias et al., 2016). Finally, *M. morganii* KT had 2 intact and 12 incomplete prophages, representing 7% of its genome sequence, which were also found in the genomes of *Providencia rustigianii, Providencia alcalifaciens, Proteus mirabilis*, and 14 other entrobacteria species (Chen et al., 2012).

Pathogenicity islands (PAIs) are DNA sequences of 10–200 Kb in size, which are widespread among bacterial pathogens and carry a number of virulence-related genes (Hacker and Kaper, 2000). In many cases the base composition of PAIs differs from host genome, allowing one to trace the origin of these elements (Schmidt and Hensel, 2004). PAIs are often located next to tRNA genes and flanked by direct repeats (Hacker and Kaper, 2000). tRNA genes are known as insertion sites for foreign DNA acquired by transformation, transduction, and conjugation (Gal-Mor and Finlay, 2006). Thus, PAIs can distribute the virulence-associated genes by horizontal gene transfer (Hochhut et al., 2006). It is known that colonization of urinary tract by UPEC strains is mediated by the expression of PAI-encoded virulence factors (Schmidt and Hensel, 2004). For example, pore-fornimg toxin α-hemolysin, which is active against majority of eukaryotic cells, is encoded by PAI (Javadi et al., 2017). Using IslandViewer4, we determined 25, 21, and 33 genomic islands (GIs) ranging from 4.2 to 34.9 Kb in the genomes of MM 1, MM 4, and MM 190, respectively. It was shown that these GIs encoded phage-associated proteins (transposase, recombinase, integrase etc.) and some toxins, such as type II toxin-antitoxin system toxins and HlyA family RTX toxin. Moreover, GIs from MM 1 include genes responsible for flagellar biosynthesis, conjugation, and arsenical resistance.

#### Hemolysins

**Figure 5:**
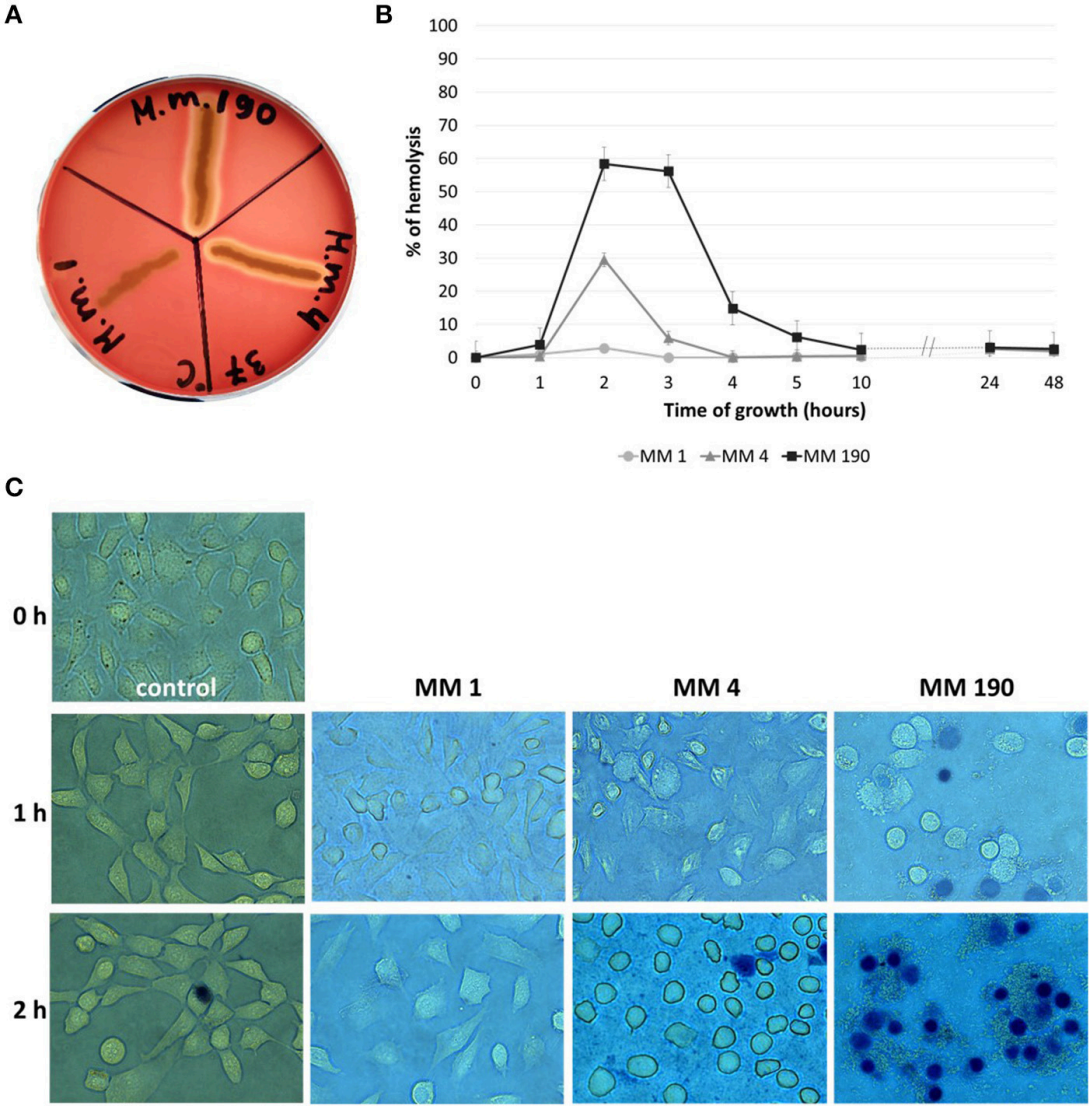
The hemolytic properties and cytotoxicity of uropathogenic *M. morganii* strains. **(A)** Evaluation of the hemolytic properties of *M. morganii* strains on 5% blood agar and **(B)** comparative analysis of hemolytic activity in 2% erythrocyte suspension during 48 h of growth. Hemolysis level in distilled water was taken as 100%. **(C)** Determination of the influence of *M. morganii* strains on T-24 bladder carcinoma cells monolayer using inverted microscopy (magnification−63x, trypan blue staining). Control–cell culture without bacteria.

Pore-forming toxins in particular hemolysins have a cytotoxic activity against broad range of cells from different species (Ristow and Welch, 2016; Lu et al., 2018). Lin et al. (1999) reported that *V. cholerae* strains express RTX toxin, which leads to the destruction of HEp-2 cells monolayer after 1 h of incubation. *M. morganii* MM 4 and *M. morganii* MM 190 were capable of β-hemolysis and their hemolytic activity was maximal at 2 h of growth (Figures 5A,B), whereas MM 190 strain showed 2 times higher hemolytic activity than MM 4. We likewise investigated the influence of *M. morganii* strains on T-24 bladder carcinoma cells monolayer. Some dead cells (stained blue) were detected in wells with MM 190 following 1 h of incubation, while 100% cell death occurred after 2 h of cultivation with the same strain (Figure 5C). MM 4 strain triggered the detachment of T-24 cells from substrate, whereas MM 1 demonstrated low cytotoxicity. These data correlate well with the hemolytic activity level of the investigated strains, suggesting a major role of secreted hemolysins from *M. morganii* in the destruction of urothelial cells.

Previously, we identified *hlyA* and *hpmA* hemolysin genes in the genomes of the *M. morganii* strains using PCR amplification (Minnullina et al., 2016). *hpmA* was common for all analyzed strains, but *hlyA* gene encoding RTX toxin was found only in MM 4 and MM 190 genomes (Table 3). It was determined that *hpmA* genes from the strains have a 97–100% identity to *M. morganii* FDAARGOS_172 and *M. morganii* KT sequences. *hlyA* genes from MM 4 and MM 190 showed 100% homology with *M. morganii* FDAARGOS_172 RTX toxin hemolysin A gene, even though homologous sequences were not identified in the genome of *M. morganii* KT.

**Table 3:** Hemolysin genes of *M. morganii* strains MM 1, MM 4, and MM 190.

**Gene**	**Product**	**Locus**		
		**MM 1**	**MM 4**	**MM 190**
*hpmA*	Filamentous hemagglutinin N-terminal domain-containing protein	DYH52_RS02020	DVJ80_RS17465	DQ401_RS10135
*hlyA*	HlyA family RTX toxin	—	DVJ80_RS01175	DQ401_RS03920

Homologs of *hpmA* and *hpmB* genes encoding calcium-independent hemolysin and hemolysin activator protein in *P. mirabilis* (Fraser et al., 2002) were previously identified in *M. morganii* KT and *M. morganii* F675 genomes (Chen et al., 2012; Olaitan et al., 2014). The genomes of all strains (MM 1, MM 4, MM 190) contained intact *hpmBA* loci encoding hemolysin activation protein and filamentous hemagglutinin N-terminal domain-containing protein. Blastx-analysis confirmed that translated nucleotide sequences of *hpmA* from MM 1, MM 4, and MM 190 only have a 38% identity with the amino acid sequence of *Proteus mirabilis* (M30186). Notwithstanding, this sequence was not determined in *Providencia* genus.

**Figure 6:**
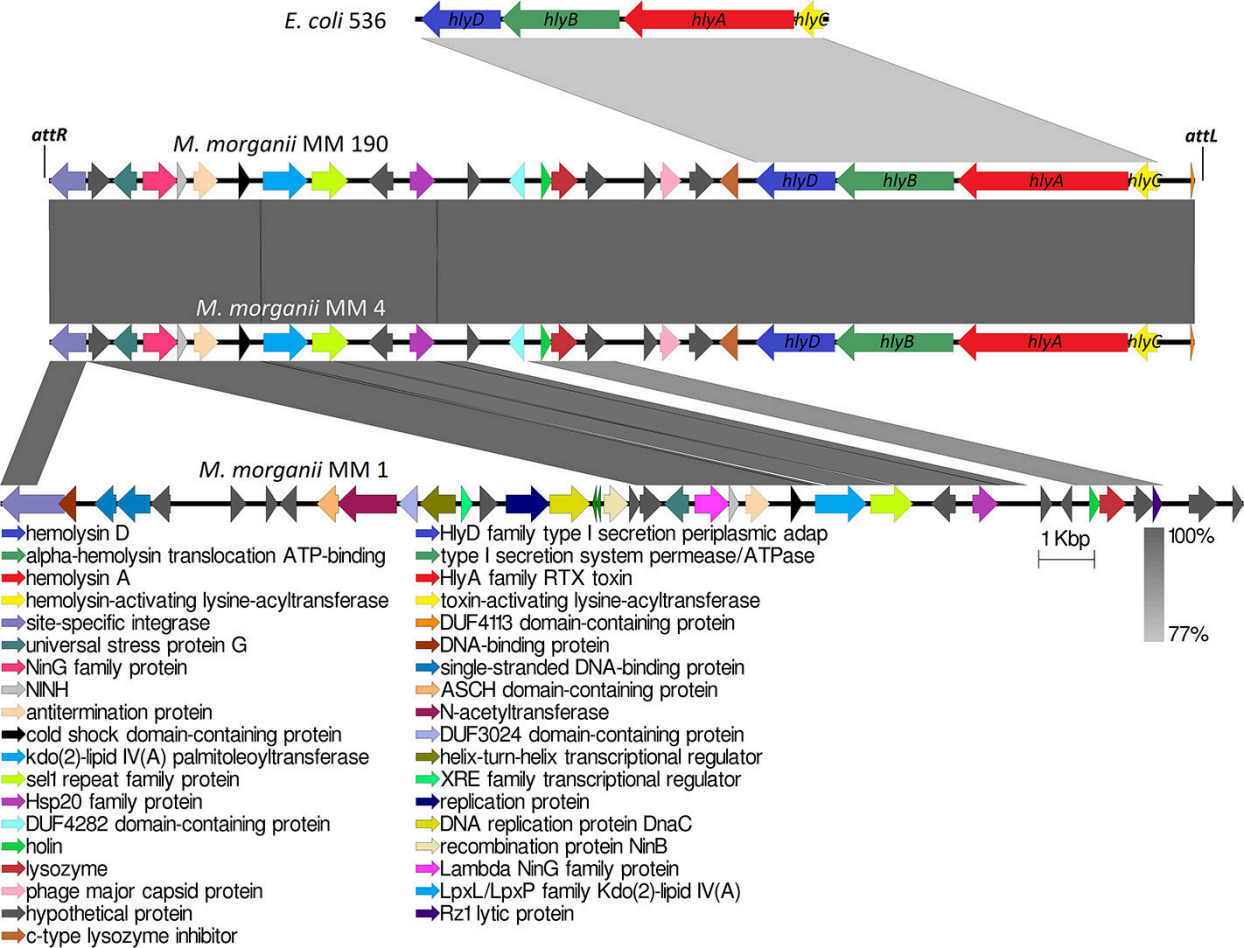
Multiple alignment of genome loci containing *hlyCABD* operon from uropathogenic *M. morganii* strains and *E. coli* 536. *attL* and *attR* indicate the attachment sites of 118970_sal4 phage.

Analogous to *M. morganii* KT isolated from blood sample, the *hlyA* gene homologous to α-hemolysin gene of *E. coli* was not detected in the genome of the non-hemolytic *M. morganii* MM 1. Genome analysis showed that only MM 4 and MM 190 strains contained intact *hlyCABD* operon having a 77% sequence homology with uropathogenic *E. coli* 536 (CP000247) (Figure 6). Translated nucleotide sequences of the RTX toxin genes had a 79% identity to amino acid sequence of *Proteus columbae* (WP_100158705). It is known that secreted hemolysin from *M. morganii* to a strong extent shares identity with the α-hemolysin of *E. coli*, showing maximal activity at the early growth hours (Gołuszko et al., 1988; Senior and Hughes, 1988). Thus, hemolytic properties of *M. morganii* MM 4 and *M. morganii* MM 190 are likely associated with expression of the *hlyA* gene. Oloomi and Bouzari (2008) reported that some strains of *E. coli* containing α-hemolysin gene did not show hemolytic properties on blood agar. Accordingly, hemolytic activity level can be associated with expression features of *hlyA* gene in different strains, since MM 4 and MM 190 hold a 100% identity in the *hlyCABD* operon.

Phage-mediated horizontal transfer of virulence-related genes is a major pathway of bacterial evolution (Javadi et al., 2017). In this connection, toxins have gained special attention among virulence factors in bacteria (Fortier and Sekulovic, 2013). A typical example is the botulinum neurotoxin (type C1), one of the most powerful toxins encoded by phage CEβ (Eklund et al., 1971). We observed that *hlyCABD* operons are inserted into genomes of *M. morganii* MM 4 and *M. morganii* MM 190 with phage sequences similar to *Salmonella* phage 118970_sal4 (dsDNA viruses, *Caudovirales* family, Figure 6). These prophages were incomplete and showed the same length (20.9 Kb) and GC content (39.3%). Meanwhile, the GC content of MM 4 and MM 190 genomes averaged at about 51%. 118970_sal4 prophage of length 42.4 Kb is well-known among *Salmonella* Heidelberg isolates. For instance, 92.3% of 196 isolates of *S*. Heidelberg contained this prophage in their genomes (Mottawea et al., 2018). However, neither the *Salmonella* spp. nor the phage 118970_sal4 genomes contained sequences homologous to RTX toxin operon.

Koronakis et al. (1987) suggested that *hlyCABD* locus of *E. coli* could have been imported from the *Proteus* genus since the GC content of its operon is about 39% (similar to genomic DNA of *Proteus* spp.) in contrast to that of the *E. coli* genome, having a 50% GC. *hlyCABD* loci from MM 4 and MM 190 strains have a GC content of 36.7% and are integrated into PAIs size of 11.3 Kb containing 9 genes. Furthermore, *hlyCABD* operon of *E. coli* 536 integrated to PAI (Hochhut et al., 2006), possesses a GC content of 40.2% in comparison with the whole genome (GC = 50.5%). It is known that many of UPEC strains contain *hlyCABD* operon integrated to PAIs, such as PAI-I, PAI-II, PAI-IV, and PAI-V. A classic illustration can be seen in *E. coli* C5, in which this sequence was located in PAI-I, which is inserted into the *leuX* loci (Javadi et al., 2017). In this connection, we suggest that *M. morganii* strains MM 4 and MM 190 could have a common source of RTX toxin operon with *E. coli* 536. Thus, our data for the first time suggest the possible transfer of *hlyCABD* operon into *M. morganii* genome by phage.

#### Ureases

**Figure 7:**
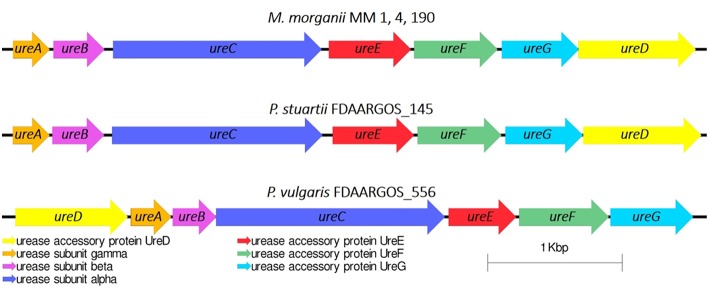
Urease gene cluster in *M. morganii* and other representatives of the *Proteeae* tribe.

Urease is a major virulence factor of uropathogenic microorganisms (Jones and Mobley, 1987), whose activity often leads to urinary stone formation (Mobley and Warren, 1987). We have investigated whether MM 1, MM 4, and MM 190 strains were capable of urease synthesis. The urease gene cluster *ureABCEFGD* was determined in their genomes in following loci: DYH52_RS16945-DYH52_RS16975 for MM 1, DVJ80_RS14270-DVJ80_RS14300 for MM 4, and DQ401_RS15300-DQ401_RS15330 for MM 190. Interestingly, the *ureR* regulatory gene was not identified in any of the genomes as well as in other *M. morganii* strains (Chen et al., 2012; Olaitan et al., 2014). It has been shown that the *ureABCEFGD* locus is conservative within the genus, the sequences show a 99–100% homology with each other and 99% with *M. morganii* KT and *M. morganii* FDAARGOS_172. Blast-analysis determined that the closest neighbors of the strains using *ureABCEFGD* locus among *Proteeae* tribe were *Providencia stuartii* FDAARGOS_145 (CP014024) and *Proteus vulgaris* FDAARGOS_556 (CP033736) with homology rates of 73 and 64%, respectively. However, in the *Proteus* genus, the *ureD* gene is located on the top of the operon, unlike in *Morganella* and *Providencia* (Figure 7).

#### Cell Capsule and Virulence Genes Regulation

**Figure 8:**
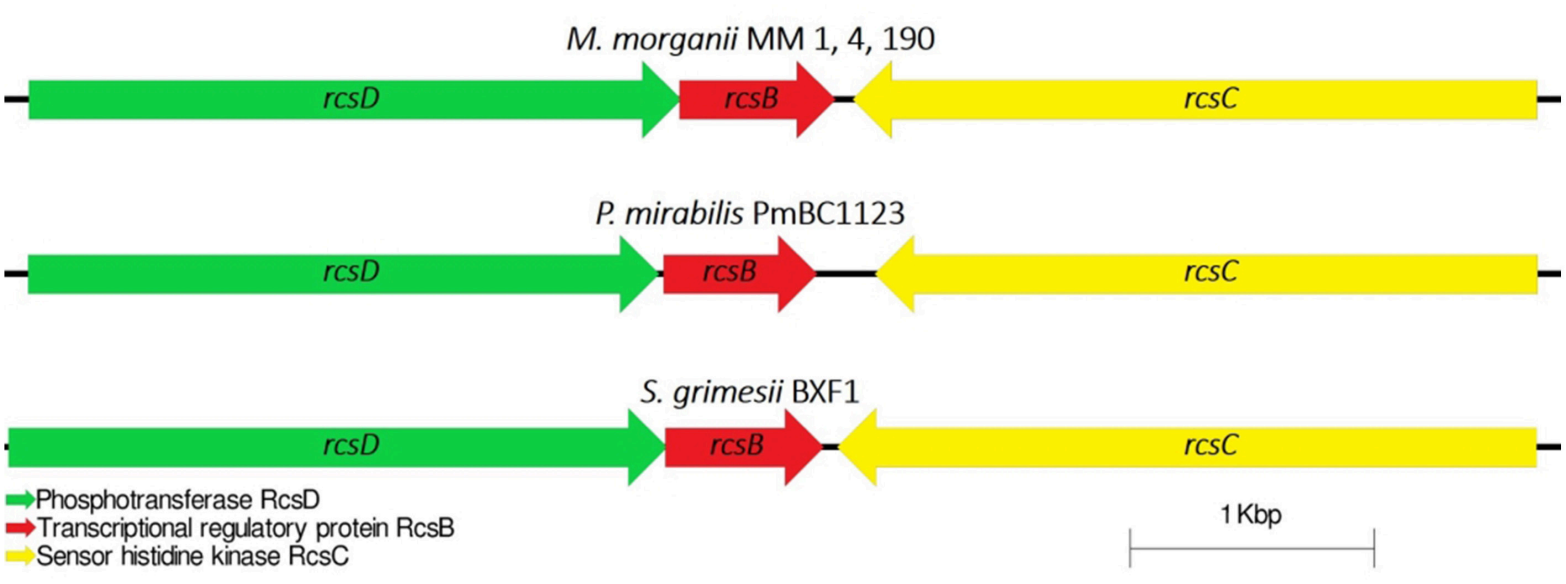
Comparative analysis of the loci of capsular synthesis regulating genes in *M. morganii, P. mirabilis*, and *Serratia grimesii*. *rcsF* gene is located distally.

Capsular polysaccharides (CPS) allow pathogenic bacteria to avoid the host immune response, mediate biofilm formation and survive in adverse conditions (Willis and Whitfield, 2013). Thirty-five genes were identified for *M. morganii* MM 1, MM 4, and MM 190 strains fitting the “capsular and extracellular polysaccharides” subcategory by the RAST server. We found that the strains contain four genes responsible for capsule synthesis regulation: *rcsB, rcsC, rcsD*, and *rcsF* (Supplementary Table 3). The Rcs phosphorelay two-component pathway initially was described as capsule and colanic acid synthesis regulator in *E. coli* (Stout, 1994). However, it was later discovered that this system plays an important role in the regulation of pathogenicity and biofilm formation (Huang et al., 2006). The Rcs phosphorelay is distributed among representatives of *Enterobacterales* order and consists of three proteins, which constitute the signaling pathway: the phosphotranspherase RcsD, the response regulator RcsB, and the hybrid kinase RcsC (Clarke, 2010). In *E. coli*, these proteins are encoded by genes which are located in convergent operons *rcsDB* and *rcsC* (Huang et al., 2006). Rcs-system is involved in the regulation of genes responsible for the synthesis of LPS, fimbriae, flagella, group 1 capsule, colanic acid, and other components of bacterial surface structures, which affect the virulence of pathogenic bacteria (Clarke, 2010; Pannen et al., 2016). *rcsDBC* sequences of MM 4 and MM 190 had a homology level of 100% to each other, and 99% with MM 1, KT, and FDAARGOS_172 strains. *M. morganii* strains exhibit different similarities with representatives of the *Proteeae* tribe relative to this locus. In particular, the RcsD, RcsB, and RcsC loci respectively showed 42, 91, and 51% amino acid sequence identity to *P. mirabilis* PmBC1123. Loci containing *rcsB, rcsC*, and *rcsD* genes were highly conserved among the other enterobacteria (Figure 8).

#### Motility and Adhesion

Expression of flagella enables UPEC to spread from the bladder to the kidneys (Wiles et al., 2008). Several reports indicated that chemotaxis may be involved in the development of diseases caused by enterobacteria (Olsen et al., 2013; Matilla and Krell, 2018). Wuichet and Zhulin (2010) reported that 54% of 450 bacterial genomes analyzed in their study contain genes associated with chemosensory pathways. Chemoreceptor genes, which have a common origin with flagellar motor and signaling genes, represent about 14 units per genome. For instance, *Borrelia burgdorferi* chemoreceptor genes comprised 5–6% of the corresponding genome (Lacal et al., 2010; Charon et al., 2012). We identified more than 60 genes associated with motility and chemotaxis in the genomes of *M. morganii* MM 1, MM 4, and MM 190 strains (Supplementary Table 3). Among them, there are genes encoding structural components of flagellum and basal body (*fli, flg, mot*), type III secretion system proteins (*ysc*), transcriptional regulators (*flh*), and chemotaxis-related genes (*che*). Flagella and chemotaxis-associated genes were located between the following loci: DYH52_RS12265-DYH52_RS12675 (MM 1), DVJ80_RS00620-DVJ80_RS00920 (MM 4), and DQ401_RS04175-DQ401_RS04475 (MM 190).

It is known that type I fimbriae allow uropathogens to colonize bladder epithelium, while mannose-resistance (MR) fimbriae or P-fimbriae promote adhesion to kidney epithelial cells (Kalita et al., 2014; Lüthje and Brauner, 2014). Seventy, Seventy-three, and Seventy-five fimbrial biogenesis and adhesion-related genes were determined in the genomes of MM 1, MM 4, and MM 190, respectively (Supplementary Table 3). Several duplicated loci containing *fim* operon encoding type I fimbriae were found in all genomes. Besides them, we detected a large *mrf* gene cluster similar to *mrp* locus of *P. mirabilis* (Meslet-Cladiere et al., 2004) and *pap* genes involved in MR-fimbriae expression, *pil* genes responsible for type IV fimbriae synthesis, and *stf* genes encoding fimbrial assembly proteins. These data confirm that the *M. morganii* strains may be important causative agents of UTIs.

## Conclusions

In this study we present the first detailed genome analysis of urine isolates of *M. morganii*. MM 1, MM 4, and MM 190 strains differed by the number of phage-associated genes. We determined that cytotoxicity and hemolytic activity of MM 4 and MM 190 strains were associated with expression of RTX toxin gene, which belongs to the genome island inserted into the genomes by a phage similar to the *Salmonella* phage 118970_sal4. Thus, the results of this study could be an important information source for investigating the pathogenesis of uropathogenic *M. morganii*.

## Data Availability

The datasets generated for this study can be found in DDBJ/EMBL/GenBank, QUOO00000000, QPLM00000000, QMKL00000000.

## Author Contributions

LM conducted the hemolysis and cytotoxicity assays, genomic DNA extraction, genome assembly and comparative analysis, and prepared the manuscript. DP performed the genome annotation and analysis. ES and LS carried out the genome sequencing of all the bacterial isolates. MS was involved in the review and design of the manuscript. AM directed conception and design of the study, wrote sections of the manuscript, and critically revised the final version of the manuscript. All authors contributed to manuscript revision, read and approved the submitted version.

### Conflict of Interest Statement

The authors declare that the research was conducted in the absence of any commercial or financial relationships that could be construed as a potential conflict of interest.
