# Supplementary material for: Comparative Genome Analysis of Uropathogenic Morganella morganii Strains
Source: Front Cell Infect Microbiol. 2019 May 22;9:167. doi: 10.3389/fcimb.2019.00167 (PMC6558430; doi:10.3389/fcimb.2019.00167)
Supplement: Supplementary file 1 [file Table_1.docx]

**Supplementary Table 1**. *M. morganii* strains with whole-genome sequences (January, 2019).

| **No.** | **Strain** | **GenBank AN** | **Host** | **Isolation source** |
| --- | --- | --- | --- | --- |
| 1. | KT | CP004345.1 | *Homo sapiens* | blood sample |
| 2. | FDAARGOS_63 | CP026046.1 | *Homo sapiens* | wound |
| 3. | FDAARGOS_172 | CP014026.2 | *Homo sapiens* | urine |
| 4. | FDAARGOS_365 | CP023505.1 | *Homo sapiens* | stool |
| 5. | KC-Tt-01 | CP025933.1 | *Tursiops truncatus* | pericardial fluid |
| 6. | AR_0057 | CP027177.1 | missing | missing |
| 7. | AR_0133 | CP028956.1 | missing | missing |
| 8. | DG56-16 | CP032295.1 | Crocodile lizard | liver |
| 9. | L241 | CP033056.1 | *Homo sapiens* | feces |
| 10. | NCTC12028 | LS483498.1 | *Homo sapiens* | stool |
| 11. | NCTC235 | LR133904.1 | missing | missing |
