# Supplementary material for: Comparative Genome Analysis of Uropathogenic Morganella morganii Strains
Source: Front Cell Infect Microbiol. 2019 May 22;9:167. doi: 10.3389/fcimb.2019.00167 (PMC6558430; doi:10.3389/fcimb.2019.00167)
Supplement: Supplementary file 2 [file Table_2.docx]

**Supplementary Table 2**. Phage insertion regions in *M. morganii* genomes.

| **Genome** | **Location** | **Length (Kb)** | **Completeness** | **GC content (%)** | **Present of *att* site** | **Score** | **Most similar phage** |
| --- | --- | --- | --- | --- | --- | --- | --- |
| MM 1 | Scaff1 | 49.7 | intact | 49.69 | + | 150 | mEp460 |
|  | Scaff1 | 34.5 | intact | 48.49 | + | 140 | phiO18P |
|  | Scaff2 | 41.3 | intact | 48.03 | + | 150 | HK446 |
|  | Scaff2 | 13.7 | incomplete | 49.80 | — | 30 | BcepB1A |
|  | Scaff3 | 20.4 | incomplete | 45.92 | + | 50 | pYD38 |
|  | Scaff3 | 25.8 | incomplete | 43.51 | + | 10 | 933W |
|  | Scaff3 | 24.5 | intact | 47.28 | — | 120 | vB_SosS_Oslo |
| MM 4 | Scaff1 | 17.3 | intact | 44.86 | — | 110 | Gifsy |
|  | Scaff1 | 20.9 | incomplete | 39.31 | + | 20 | 118970_sal4 |
|  | Scaff1 | 13.7 | incomplete | 49.79 | — | 30 | BcepB1A |
|  | Scaff2 | 33.8 | intact | 46.80 | + | 130 | phiO18P |
|  | Scaff9 | 19.2 | intact | 49.97 | — | 140 | cdtl |
|  | Scaff15 | 12 | intact | 53.03 | — | 130 | 118970_sal3 |
| MM 190 | Scaff1 | 48.8 | questionab | 48.27 | + | 90 | phiV10 |
|  | Scaff1 | 35.3 | intact | 44.24 | — | 150 | Fels1 |
|  | Scaff1 | 13.7 | incompl | 49.79 | — | 30 | BcepB1A |
|  | Scaff1 | 20.9 | incompl | 39.32 | + | 20 | 118970_sal4 |
|  | Scaff2 | 16.4 | incompl | 49.16 | — | 30 | SfV |
|  | Scaff2 | 14.8 | intact | 46.91 | + | 100 | ST64B |
|  | Scaff8 | 25.7 | incompl | 48.66 | + | 10 | 118970_sal3 |
|  | Scaff8 | 45.2 | intact | 47.53 | + | 110 | ENT47670 |
|  | Scaff12 | 19 | intact | 49.89 | — | 140 | 118970_sal3 |
|  | Scaff18 | 7.7 | intact | 53.40 | — | 110 | HK446 |
