# Supplementary material for: Comparative Genome Analysis of Uropathogenic Morganella morganii Strains
Source: Front Cell Infect Microbiol. 2019 May 22;9:167. doi: 10.3389/fcimb.2019.00167 (PMC6558430; doi:10.3389/fcimb.2019.00167)
Supplement: Supplementary file 3 [file Table_3.docx]

**Supplementary Table 3**.Viruence-related genes of uropathogenic *M. morganii* strains.

| **Category** | **MM 1** | | **MM 4** | | **MM 190** | |
| --- | --- | --- | --- | --- | --- | --- |
|  | **No. of genes** | **Locus** | **No. of genes** | **Locus** | **No. of genes** | **Locus** |
| Fimbriae and adhesins  (MS, MR, type IV fimbriae, adhesins,  secretion system proteins  etc.) | 70 | DYH52_RS01195-DYH52_RS01205,  DYH52_RS03770,  DYH52_RS03815-DYH52_RS03910,  DYH52_RS03965-DYH52_RS03980,  DYH52_RS04460-DYH52_RS04485,  DYH52_RS04580,  DYH52_RS05455,  DYH52_RS05460,  DYH52_RS05720-DYH52_RS05735,  DYH52_RS11255-DYH52_RS11310,  DYH52_RS14560,  DYH52_RS14585,  DYH52_RS16240,  DYH52_RS16495,  DYH52_RS16500,  DYH52_RS17235-DYH52_RS17255,  DYH52_RS17520-DYH52_RS17535,  DYH52_RS17635,  DYH52_RS17650, DYH52_RS17895 | 73 | DVJ80_RS01445,  DVJ80_RS03400,  DVJ80_RS03405,  DVJ80_RS05635,  DVJ80_RS05640,  DVJ80_RS05725-DVJ80_RS05755,  DVJ80_RS05950,  DVJ80_RS06225-DVJ80_RS06240,  DVJ80_RS06295-DVJ80_RS06390,  DVJ80_RS06440,  DVJ80_RS07640,  DVJ80_RS08290,  DVJ80_RS08315,  DVJ80_RS10230-DVJ80_RS10250,  DVJ80_RS10980,  DVJ80_RS12155-DVJ80_RS12165,  DVJ80_RS12540,  DVJ80_RS14525,  DVJ80_RS14745-DVJ80_RS14760,  DVJ80_RS15005,  DVJ80_RS15295-DVJ80_RS15310,  DVJ80_RS15575-DVJ80_RS15595,  DVJ80_RS15840,  DVJ80_RS15845,  DVJ80_RS16110-DVJ80_RS16125,  DVJ80_RS16740 | 75 | DQ401_RS01455,  DQ401_RS03410,  DQ401_RS04710,  DQ401_RS05560-DQ401_RS05580,  DQ401_RS06310,  DQ401_RS07830,  DQ401_RS07835,  DQ401_RS07920-DQ401_RS07950,  DQ401_RS08420-DQ401_RS08435,  DQ401_RS08490-DQ401_RS08585,  DQ401_RS08635,  DQ401_RS10685,  DQ401_RS10710,  DQ401_RS12620-DQ401_RS12630,  DQ401_RS14595,  DQ401_RS14840-DQ401_RS14855,  DQ401_RS15075,  DQ401_RS15660-DQ401_RS15675,  DQ401_RS15775-DQ401_RS15790,  DQ401_RS16055-DQ401_RS16075,  DQ401_RS16710,  DQ401_RS17415,  DQ401_RS17605,  DQ401_RS17610,  DQ401_RS17875-DQ401_RS17890 |
| Motility  (flagellar proteins, T3SS components, transcriptional regulators etc.) | 60 | DYH52_RS02520,  DYH52_RS03040,  DYH52_RS04050,  DYH52_RS04155-DYH52_RS04170,  DYH52_RS04190,  DYH52_RS11330,  DYH52_RS12265-DYH52_RS12335,  DYH52_RS12350-DYH52_RS12370,  DYH52_RS12380-DYH52_RS12415,  DYH52_RS12430-DYH52_RS12495,  DYH52_RS12645-DYH52_RS12675,  DYH52_RS16055,  DYH52_RS17890,  DYH52_RS18210 | 63 | DVJ80_RS00620-DVJ80_RS00690,  DVJ80_RS00705-DVJ80_RS00770,  DVJ80_RS00785-DVJ80_RS00850,  DVJ80_RS00890-DVJ80_RS00920,  DVJ80_RS06015,  DVJ80_RS06030-DVJ80_RS06050,  DVJ80_RS06155,  DVJ80_RS10205,  DVJ80_RS13805,  DVJ80_RS13940,  DVJ80_RS14095,  DVJ80_RS15055,  DVJ80_RS16745,  DVJ80_RS17805 | 65 | DQ401_RS04175-DQ401_RS04205,  DQ401_RS04245-DQ401_RS04310,  DQ401_RS04325-DQ401_RS04390,  DQ401_RS04405-DQ401_RS04475,  DQ401_RS05535,  DQ401_RS08210,  DQ401_RS08225-DQ401_RS08245,  DQ401_RS08350,  DQ401_RS09055,  DQ401_RS14270,  DQ401_RS14400,  DQ401_RS14405,  DQ401_RS15510,  DQ401_RS15535,  DQ401_RS17420,  DQ401_RS18125 |

| Toxins  (hemolysins, hemagglutinins, insecticidal toxins, secretion system proteins  etc.) | 30 | DYH52_RS02010-  DYH52_RS02025,  DYH52_RS02050,  DYH52_RS02160,  DYH52_RS02165,  DYH52_RS02870,  DYH52_RS03715,  DYH52_RS04415,  DYH52_RS06560,  DYH52_RS09205,  DYH52_RS09210,  DYH52_RS09485,  DYH52_RS11500,  DYH52_RS11655,  DYH52_RS12340,  DYH52_RS12345,  DYH52_RS14045,  DYH52_RS14380,  DYH52_RS14385,  DYH52_RS14405,  DYH52_RS14415,  DYH52_RS14800,  DYH52_RS15150,  DYH52_RS15885,  DYH52_RS15940,  DYH52_RS15945,  DYH52_RS15965,  DYH52_RS16640 | 32 | DVJ80_RS00025,  DVJ80_RS00055,  DVJ80_RS00695,  DVJ80_RS01165-DVJ80_RS01180,  DVJ80_RS04055,  DVJ80_RS04060,  DVJ80_RS04275,  DVJ80_RS05255,  DVJ80_RS05800,  DVJ80_RS07190,  DVJ80_RS07890,  DVJ80_RS08075,  DVJ80_RS08465,  DVJ80_RS08475,  DVJ80_RS08495,  DVJ80_RS08500,  DVJ80_RS08835,  DVJ80_RS09890,  DVJ80_RS10035,  DVJ80_RS13135,  DVJ80_RS13145,  DVJ80_RS13850,  DVJ80_RS14605,  DVJ80_RS16360,  DVJ80_RS17460,  DVJ80_RS17465,  DVJ80_RS17470,  DVJ80_RS17475,  DVJ80_RS18145 | 31 | DQ401_RS00790,  DQ401_RS00795,  DQ401_RS03630,  DQ401_RS03635,  DQ401_RS03640,  DQ401_RS03915-DQ401_RS03930,  DQ401_RS04400,  DQ401_RS05040,  DQ401_RS05075,  DQ401_RS07450,  DQ401_RS07995,  DQ401_RS09490,  DQ401_RS09995,  DQ401_RS10000,  DQ401_RS10130-DQ401_RS10145,  DQ401_RS10470,  DQ401_RS10870,  DQ401_RS10890,  DQ401_RS10895,  DQ401_RS11230,  DQ401_RS13600,  DQ401_RS13610,  DQ401_RS14315,  DQ401_RS14995,  DQ401_RS17035 |
| --- | --- | --- | --- | --- | --- | --- |

| Biofilm formation (AI-2, biofilm formation regulators) | 9 | DYH52_RS08815,  DYH52_RS08825-DYH52_RS08850,  DYH52_RS09170,  DYH52_RS12205 | 8 | DVJ80_RS00565,  DVJ80_RS01995-DVJ80_RS02030 | 11 | DQ401_RS02825-DQ401_RS02860,  DQ401_RS03160,  DQ401_RS04530,  DQ401_RS10475 |
| --- | --- | --- | --- | --- | --- | --- |
| Ureases | 7 | DYH52_RS16945-DYH52_RS16975 | 7 | DVJ80_RS14270-DVJ80_RS14300 | 7 | DQ401_RS15300-DQ401_RS15330 |
| Capsule synthesis regulation | 4 | DYH52_RS12795-DYH52_RS12805,  DYH52_RS16225 | 4 | DVJ80_RS01040-DVJ80_RS01050,  DVJ80_RS15020 | 4 | DQ401_RS04045-DQ401_RS04055,  DQ401_RS14580 |
| Cell invasion | 3 | DYH52_RS04140,  DYH52_RS04330,  DYH52_RS04340 | 1 | DVJ80_RS06060 | 1 | DQ401_RS08255 |
