# Supplementary material for: Comparative Genome Analysis of Uropathogenic Morganella morganii Strains
Source: Front Cell Infect Microbiol. 2019 May 22;9:167. doi: 10.3389/fcimb.2019.00167 (PMC6558430; doi:10.3389/fcimb.2019.00167)
Supplement: Supplementary file 4 [file Data_Sheet_1.docx]

**Codes used to pre-process and assemble the genomes of *M. morganii***

**Filtration**

Command line: java -jar trimmomatic-0.36. jar SE -phred33 reads_R2.fastq.gz reads_R2_filtrated.fq CROP:244 HEADCROP:5

**Genome assembly**

Command line: spades.py -1 reads_R1.fq -2 reads_R2.fq --careful -o output_dir -t8 -m256

System information:

SPAdes version: 3.10.0

Python version: 2.7.12

OS: Linux-4.4.0-103-generic-x86_64-with-Ubuntu-16.04-xenial

Mode: read error correction and assembling

Debug mode is turned OFF
