# Supplementary figures and images for: Comparative Genome Analysis of Uropathogenic Morganella morganii Strains
Source: Front Cell Infect Microbiol. 2019 May 22;9:167. doi: 10.3389/fcimb.2019.00167 (PMC6558430; doi:10.3389/fcimb.2019.00167)

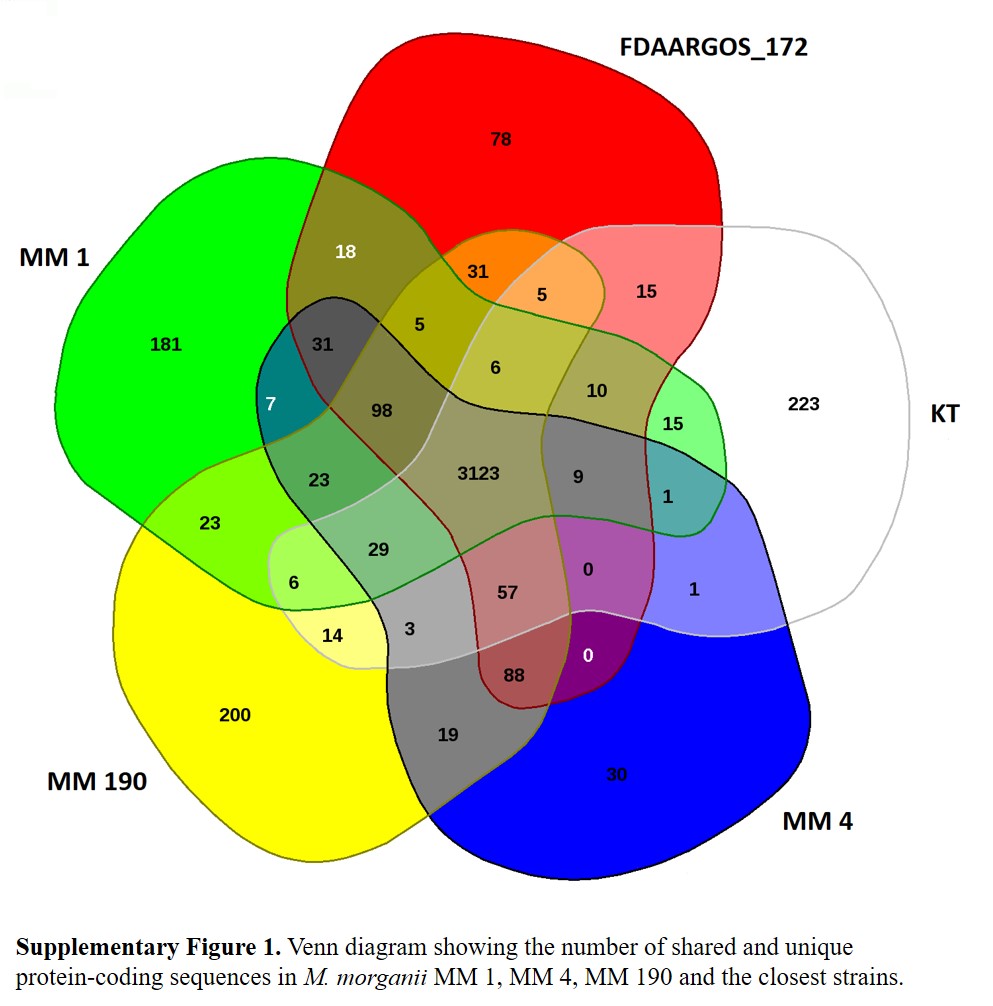

Supplement: Supplementary file 5 [file Image_1.JPEG]

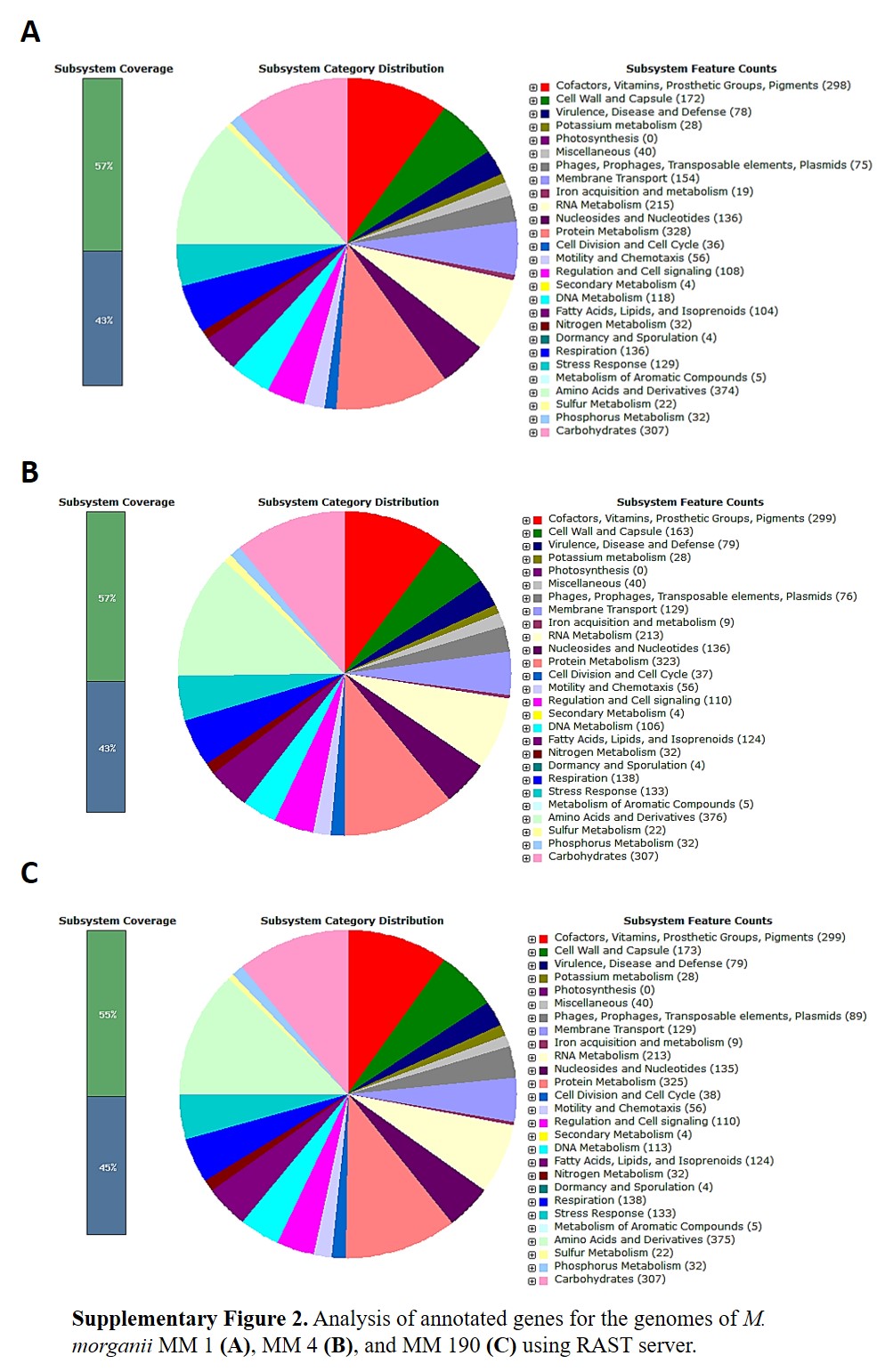

Supplement: Supplementary file 6 [file Image_2.JPEG]
